# Cost-effectiveness of smoking cessation to prevent age-related macular degeneration

**DOI:** 10.1186/1478-7547-6-18

**Published:** 2008-09-11

**Authors:** Susan F Hurley, Jane P Matthews, Robyn H Guymer

**Affiliations:** 1 Bainbridge Consultants, 222/299 Queen St, Melbourne, VIC 3000, Australia; 2 School of Medicine, Griffith University; 3 School of Population Health, The University of Melbourne; 4 Macular Research Unit, Department of Ophthalmology, Centre for Eye Research Australia, The University of Melbourne

## Abstract

**Background:**

Tobacco smoking is a risk factor for age-related macular degeneration, but studies of ex-smokers suggest quitting can reduce the risk.

**Methods:**

We fitted a function predicting the decline in risk of macular degeneration after quitting to data from 7 studies involving 1,488 patients. We assessed the cost-effectiveness of smoking cessation in terms of its impact on macular degeneration-related outcomes for 1,000 randomly selected U.S. smokers. We used a computer simulation model to predict the incidence of macular degeneration and blindness, the number of quality-adjusted life-years (QALYs), and direct costs (in 2004 U.S. dollars) until age 85 years. Cost-effectiveness ratios were based on the cost of the Massachusetts Tobacco Control Program. Costs and QALYs were discounted at 3% per year.

**Results:**

If 1,000 smokers quit, our model predicted 48 fewer cases of macular degeneration, 12 fewer cases of blindness, and a gain of 1,600 QALYs. Macular degeneration-related costs would decrease by $2.5 million if the costs of caregivers for people with vision loss were included, or by $1.1 million if caregiver costs were excluded. At a cost of $1,400 per quitter, smoking cessation was cost-saving when caregiver costs were included, and cost about $200 per QALY gained when caregiver costs were excluded. Sensitivity analyses had a negligible impact. The cost per quitter would have to exceed $77,000 for the cost per QALY for smoking cessation to reach $50,000, a threshold above which interventions are sometimes viewed as not cost-effective.

**Conclusion:**

Smoking cessation is unequivocally cost-effective in terms of its impact on age-related macular degeneration outcomes alone.

## Background

There is a strong association between tobacco smoking and age-related macular degeneration.[1] A pooled analysis of data from the 3 largest population-based prevalence surveys found risks for current smokers relative to never smokers were 4.55-fold higher for neovascular age-related macular degeneration and 2.54-fold higher for geographic atrophy.[2] These relative risks were approximately halved in ex-smokers, suggesting that the adverse effect of smoking is reversible.[1,2] Despite these findings, the management of macular degeneration has focused on treatment rather than prevention.

Previous analyses of the economics of smoking cessation have considered the improved quality of life, increased life expectancy and lower health care expenditures associated with the reduced incidence of illnesses such as cardiovascular disease, stroke, lung cancer and chronic obstructive pulmonary disease. [3-5] These analyses have found that interventions that encourage and facilitate quitting are very cost-effective, with net cost savings in some instances.[5] However the economic impact of quitting on macular degeneration has not been analysed.

The purpose of the present analysis was therefore to quantify the health and health economic benefits of smoking cessation in relation to age-related macular degeneration alone. We estimated the cost-effectiveness of a tobacco control program in terms of prevention of blindness and improvement in quality of life as a consequence of the decreased risk of macular degeneration. We based our estimates of the cost of quitting on the Massachusetts Tobacco Control program conducted in the 1990s, and which had the highest per capita expenditure on tobacco control in the world.[6] Our analyses investigated the extent to which the cost of such smoking cessation programs will be offset by savings in the cost of care and medical treatment due to prevention of vision loss.

## Methods

### Model Overview

We developed a Markov model to simulate the risk and progression of macular degeneration in cigarette smokers and quitters in the United States, my modifying a Markov model we had published previously.[7] The previous model was designed to assess the cost-effectiveness of ranibizumab, a new treatment for the neovascular form of macular degeneration. Both it, and the model reported here, were programmed using the decision analysis software TreeAge.[8] The smoking and macular degeneration model predicted the following outcomes for smokers and quitters: the probability of developing macular degeneration, the probability of blindness (defined as visual acuity < 35 letters read on the logMAR chart, or Snellen equivalent < 20/200),[9] the number of years spent blind (blind-years), the number of quality-adjusted life-years (QALYs), and direct costs (excluding patient time and travel costs) from a societal perspective in 2004 U.S. dollars.

The Markov model tracked subjects in each 5-year age group from 15–19 years for the remainder of their lifetime, censored at age 85 years. Each year, subjects were at risk of developing either the neovascular or the geographic atrophy form of macular degeneration, or dying. The neovascular form (or "wet" age-related macular degeneration) involves serous or haemorrhagic detachment of the retinal pigment epithelium or sub-retinal pigment epithelial haemorrhages. Geographic atrophy (or "dry" age-related macular degeneration) involves a discrete area of retinal depigmentation with a sharp border and visible choroidal vessels. Disease progression for subjects who developed macular degeneration was characterized by a series of annual transitions between health states, defined by visual acuity, as described in our previous paper.[7] Briefly, the five health states considered corresponded to the number of letters read on the log-MAR chart[9] being > 85, 70–80, 55–65, 40–50, and < 35 (blind). We assumed that, each year, a patient's visual acuity would increase by 15 letters, remain the same, decrease by 15 letters, or decrease by 30 letters. We assumed that smoking cessation decreased the risk of developing macular degeneration and the risk of death from all causes, but did not affect disease progression. For each 5-year age-group, for males and females separately, the course of 10,000 smokers was simulated one at a time, firstly assuming that each subject continued to smoke, then assuming that all subjects quit.

We assessed the macular degeneration-related benefits of smoking cessation by comparing outcomes for a hypothetical cohort of 1,000 smokers. This cohort was randomly selected, stratified by 5-year age-group and sex, from a population simulated to represent the U.S. population of smokers in 2004.[10] Cost-effectiveness ratios were estimated using data on the cost of smoking cessation from the comprehensive Massachusetts Tobacco Control Program, conducted in the 1990s.[6] Future costs, blind-years and QALYs were discounted at 3% per year.[11]

### Estimates for model variables

#### Incidence of age-related macular degeneration in smokers

Annual incidence probabilities for each form of age-related macular degeneration for the general U.S. population (i.e. for smokers, ex-smokers and never-smokers combined) were based on the 5-year incidence of late age-related maculopathy in the Beaver Dam Eye Study,[12] and the proportions of geographic atrophy and neovascular age-related macular degeneration estimated in a pooled analysis of incidence studies from the U.S., the Netherlands and Australia.[13] We used a method previously described [4] to estimate probabilities for smokers from these population probabilities. Briefly, the population probabilities were adjusted on the basis of 2004–2005 smoking prevalence in the U.S.,[10] and the relative risks of each type of age-related macular degeneration in smokers and ex-smokers estimated from pooled incidence data (see Table 1).[2]

**Table 1:** Annual incidence probabilities of age-related macular degeneration for current smokers and ex-smokers who quit 15 years previously*

**Type of age-related macular degeneration and age range**	**Smoker (per 1000)^†^**	**Quitter (per 1000)^‡^**
**Neovascular**		
< 55 years	0.00	0.00
55–64 years	0.86	0.73
65–74 years	4.50	3.84
75–84 years	20.61	17.60

**Geographic atrophy**		
< 55 years	0.00	0.00
55–64 years	0.39	0.23
65–74 years	1.86	1.11
75–84 years	8.09	4.83

* Men and women combined

^† ^Estimated[4] from Beaver Dam Eye Study incidence data,[12] U.S. smoking prevalence in 2004–2005[10] and the relative risks of age-related macular degeneration for smokers and ex-smokers relative to never-smokers.[2]

^‡ ^Estimated according to the relative risk functions plotted in Figure 2.

#### Incidence of age-related macular degeneration in quitters

Through a comprehensive MEDLINE search combining the search terms "smoking" and "macular degeneration", we identified 7 studies that reported the risk of age-related macular degeneration for ex-smokers by time since quitting: 2 prospective cohort studies,[14,15] 2 case-control studies[16,17], and 3 cross-sectional studies. [18-20] These studies analysed the smoking profiles of a total of 1,488 people with age-related macular degeneration. We extracted data on the relative risk of age-related macular degeneration for ex-smokers relative to never-smokers and the time after quitting that each risk was assessed. In the study by Seddon et al.[15] risks were reported relative to current-smokers, and we therefore divided them by the risks for never-smokers relative to current-smokers to obtain risks for ex-smokers relative to never smokers. Where time was reported in the publication as a range, we took the time since quitting to be the midpoint; for example, < 20 years and 5 – 14 years were recorded as 10 years. Where time was reported as "greater than" or "equal or greater than" a specified number of years, we took the time since quitting to be the specified time plus 10 years, so > 20 years and ≥ 20 years were recorded as 30 years.

We assumed the following model for the risk, RR(t), of age-related macular degeneration for ex-smokers relative to never-smokers:

RR(t) = [(RR_0_- 1)]e^-t/τ ^+ 1

where:

RR_0 _was the relative risk of developing age-related macular degeneration for current-smokers versus never-smokers

t was the time, in months, since quitting

τ was a slope parameter that was inversely proportional to the rate at which the relative risk decreased with time since quitting.

We assumed that the asymptotic value of the relative risk, RR(∞), was 1, i.e., that the risk of developing macular degeneration for quitters eventually equalled the risk for never-smokers. The data from the 7 studies were consistent with this assumption. Six of the 7 studies had a RR measured or inferred at 30 years, and values ranged from 0.85 to 1.5. We assumed that RR_0 _depended on the particular study population and the type of macular degeneration (neovascular or geographic atrophy). A separate value for RR_0 _was therefore estimated for each study. However, due to the paucity of data, we assumed that the parameter τ did not depend on age, or sex, or the type of macular degeneration.

The values of RR_0 _and τ were estimated by fitting the non-linear model:

ln(RR(t)) = ln([(RR_0_- 1)]e^-t/τ ^+ 1) + ε

where the regression errors (ε) were assumed to be independent.

The analyses were carried out using the non-linear regression procedure (Levenberg-Marquardt estimation method) in the SPSS software package. The natural logarithms of the relative risks were weighted proportional to the inverse of their variances, which were estimated from the reported confidence intervals for the relative risks. As confidence intervals were unavailable for the relative risks calculated from the data reported by Seddon and colleagues,[15] the variances of the natural logarithms of the relative risks were conservatively estimated by summing the variances of the natural logarithms of the risks of ex-smokers relative to current-smokers and those of never-smokers relative to current-smokers.[21]

**Figure 1:**
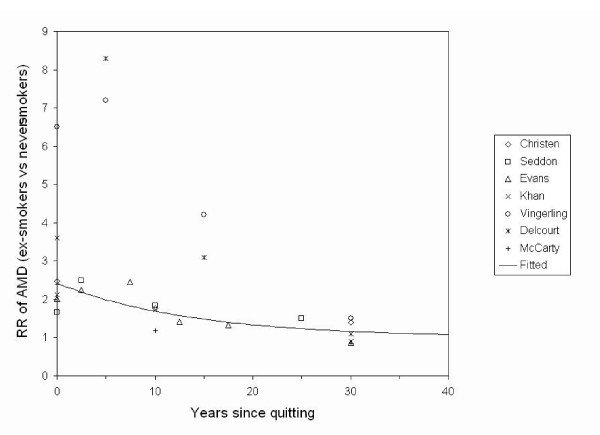
Risk over time of age-related macular degeneration (AMD) for ex-smokers versus never-smokers.

The estimated value of τ was 165, with an asymptotic standard error of 35, and 95% Confidence Interval of 90 – 241. The model therefore predicted that every 9.5 years (95% confidence interval: 5.2 – 13.9 years)the difference between a quitter's risk of age-related macular degeneration and that of a never-smoker will be halved, because when t = ln(2)*τ, (RR(t)-1) = (RR_0 _- 1)/2^k^, where k is an arbitrary integer. The data and the predicted decline over time in risk of age-related macular degeneration for ex-smokers relative to never-smokers are plotted in Figure 1. For illustrative purposes, a common value of RR_0 _was assumed for the fitted model, estimated by pooling the data. A number of alternative models were run, excluding observations in the first 10 years to mitigate any "sick quitter" effect, and considering studies with younger subjects and older subjects separately. These alternative models gave values of τ within the 95% confidence interval for τ in the base model.

In our Markov model of the impact of smoking cessation on age-related macular degeneration, we needed estimates of the risks, for ex-smokers relative to current-smokers, over time since quitting.

We therefore used the model parameter estimate that describes the rate of decline in the relative risk, and the risks of each type of macular degeneration for smokers relative to never-smokers[2] to calculate the risk, each year after quitting, of neovascular macular degeneration and geographic atrophy, for an ex-smoker relative to a current smoker, using the following formulae.

By definition:

rr(t) = RR(t)/RR_0_

or

rr(t) = [(1-1/RR_0_)]e^-t/τ ^+ 1/RR_0_.

The values of RR_0 _(the risks of macular degeneration for current smokers relative to never smokers) for neovascular disease and geographic atrophy were assumed to be 4.55 (asymptotic 95% CI: 2.74 – 7.54) and 2.54 (asymptotic 95% CI: 1.25 – 5.17), respectively.[2]

Therefore, for neovascular age-related macular degeneration:

rr(t) = 0.220 e^-t/165 ^+ 0.780

and, for geographic atrophy:

rr(t) = 0.606 e^-t/165 ^+ 0.394

**Figure 2:**
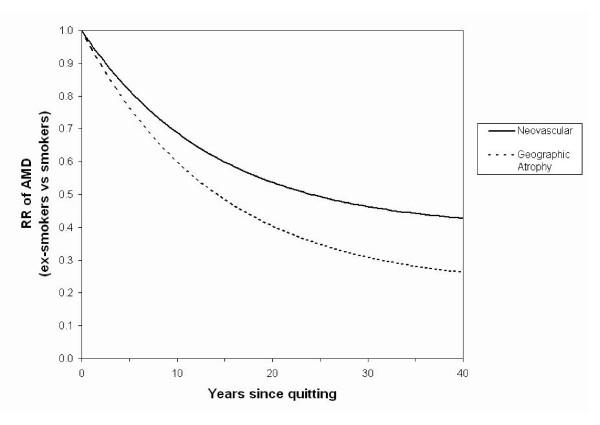
Predicted declines over time after smoking cessation in the Relative Risk (RR) of neovascular age-related macular degeneration (AMD) and geographic atrophy, for ex-smokers compared with smokers.

The predicted declines in these risks over time are shown in Figure 2. The incidence probabilities for smokers were multiplied by these relative risks to obtain incidence probabilities for ex-smokers, and such probabilities 15 years after quitting are presented in Table 1.

#### Progression, treatment and costs of age-related macular degeneration

Our assumptions about the distribution of visual acuity at diagnosis of age-related macular degeneration, disease progression, treatment and costs came from a previous paper in which we analysed the cost-effectiveness of ranibizumab, a new treatment for the neovascular form of macular degeneration.[7]

We assumed that 90% of patients with the neovascular form of disease were treated with ranibizumab, that ranibizumab's cost was the current wholesale price ($1,950 per dose)[22]and that its effectiveness and dosing regimen corresponded to the base-case scenario described in the previous paper,[7] i.e. it was effective for 4 years, during which time it was given monthly for the first 2 years then 3 monthly. Costs for geographic atrophy-related medical care (which were not considered in the ranibizumab cost-effectiveness analysis) were sourced from Halpern and colleagues' analyses of Medicare files.[23] We converted the average annual cost for patients with "dry only" disease ($345 in 2001 dollars) to 2004 U.S. dollars ($395) using the medical care Consumer Price Index.[24]

#### Utilities

We assumed vision loss was associated with reduced quality of life, and used the visual acuity-specific utility estimates from patients with age-related macular degeneration sourced for the ranibizumab cost-effectiveness analysis.[7,25] We assumed there was no reduction in utility associated with smoking or quitting.

#### Probabilities of death for smokers and quitters

We used the method previously described, [4] and referred to above for the incidence of macular degeneration in smokers, to estimate probabilities of death for smokers from all causes mortality data for the general U.S. population in 2004.[26] Smoking prevalence in 2004–5[10] and relative risks of all causes mortality for smokers from the American Cancer Society (ACS) Cancer Prevention Study (CPS-II) the U.S.[27] were used in the calculations.

Quitters' mortality probabilities were estimated by applying a function that described the decline in the risk of death from all causes for quitters relative to smokers[4] to the probability of death for smokers. The function was based on data from the ACS CPS-II. [27]

#### Cost per quitter

The Massachusetts tobacco control program started in 1993, and spent over $200 million by 1999 on interventions including a mass media campaign, services such as treatment and telephone counselling to help smokers quit, and promotion of local policies.[6] By 1999, the adult smoking prevalence in Massachusetts was 3.9% lower than in 48 other U.S. states without such programs.[6,28] This represented about 183,600 fewer adult smokers, based on the number of people aged 18 and over in Massachusetts in 1999.[29] The cost per quitter was therefore assumed to be $1,400 after adjusting the cost of the program (assumed to be in 1995 U.S. dollars) to 2004 dollars on the basis of the Consumer Price Index.[30]

### Sensitivity analyses

We performed sensitivity analyses to investigate the impact of key model assumptions on QALYs, costs and the incremental cost per QALY gained. The parameter that describes the rate of decline in risk of macular degeneration after quitting was varied from its low to its high 95% confidence limit, and different assumptions about the disutility of vision loss and treatment of neovascular age-related macular degeneration[7] were investigated. A threshold analysis was conducted to determine the cost per quitter that gave a cost per QALY for smoking cessation of $50,000.

## Results

The expected lifetime macular degeneration-related health outcomes for 1000 randomly selected smokers, who either continued to smoke or quit, are summarized in Table 2. Our model predicted that quitters would have 48 fewer cases of macular degeneration than continuing smokers, leading to 12 fewer cases of blindness, 21 fewer blind-years and 1,611 more QALYs.

**Table 2:** Expected Lifetime* AMD-related^† ^Health Outcomes for 1,000 Randomly Selected Smokers,^‡ ^who either Continue Smoking or Quit.

**AMD-related Health Outcomes**	**Continuing Smokers**		**Quitters**		**Benefits of quitting**	
	Mean	s.e	Mean	s.e	Mean	s.e
	
Cases of AMD						
Neovascular	86	0.6	45	0.4	-41	0.7
Geographic Atrophy	34	0.4	27	0.3	-7	0.5
Total	120	0.7	72	0.5	-48	0.9
Cases of blindness^§^	32	0.4	20	0.3	-12	0.5
Blind-years	75	1.2	54	1.1	-21	1.6
QALYs	19,168	10	20,778	9	1,611	14

s.e = standard error

* Censored at age 85 years

^† ^AMD: Age-related macular degeneration

^‡ ^From the U.S. population of smokers in 2004–2005[10]

^§ ^Visual acuity ≤ 20/200 (logMAR equivalent ≤ 35 letters)

The lifetime macular degeneration-related costs and cost-effectiveness ratios associated with smoking cessation are summarized in Table 3. When the costs of caregivers for people with macular degeneration and vision loss were included in the analysis, the costs for 1000 quitters were about $2.52 million lower than those for 1000 continuing smokers. At a cost per quitter of $1,400, quitting was "dominant" in terms of macular degeneration outcomes alone, i.e. it was both cost saving and improved health. Although quitting was no longer dominant when the cost of caregiving was excluded from the analysis, the incremental cost per QALY gained through quitting was only $197.

**Table 3:** Expected Lifetime* AMD-related† Costs for 1,000 Randomly Selected Smokers^‡ ^who either Continue Smoking or Quit, and Cost-Effectiveness Ratios for a Tobacco Control program.^§^.

**Cost assumptions**	**Lifetime AMD-related costs**			**Cost-effectiveness Ratios (assuming a cost per quitter of $1,400)**		
	**Continuing Smokers**	**Quitters**	**Benefit of quitting**	**Cost per case of blindness prevented**	**Cost per blind-year prevented**	**Cost per QALY gained**
	**$ ** **mean (s.e)**	**$** **mean (s.e)**	**$** **mean (s.e)**	**$**	**$**	**$**

**Including caregiver costs**	7,810,000 (73,080)	5,286,000 (64,520)	-2,523,000 (97,490)	Dominant^¶^	Dominant	Dominant
**Excluding caregiver costs**	2,786,000 (19,830)	1,703,000 (15,930)	-1,082,000 (25,440)	26,500	15,142	197

s.e = standard error

* Censored at age 85 years

^† ^AMD: Age-related macular degeneration

^‡ ^From the U.S. population of smokers in 2004–2005[10]

^§^Costs are in 2004 U.S. dollars and were rounded. Costs, blind-years and QALYs were discounted at 3% per annum

¶ Dominant: Quitting improved health outcomes and was cost saving.

In the sensitivity analyses, quitting smoking remained dominant under all assumptions tested, when caregiver costs were included in the analyses and the cost per quitter was $1,400. The sensitivity analyses excluding caregiver costs are summarized in Table 4. The cost-effectiveness ratios were all still considerably less than $1,000 per QALY. The cost per quitter had to exceed $77,000 for the incremental cost per QALY associated with smoking cessation to reach $50,000.

**Table 4:** Sensitivity Analyses of the Lifetime* AMD-related^† ^Benefits of Quitting for 1,000 Randomly Selected Smokers^‡^, and Cost-Effectiveness of a Tobacco Control Program.^§^.

**Model Variable**	**Lifetime AMD-related Benefits of Quitting**		**Cost per QALY gained (assuming a cost per quitter of $1,400)**
	**QALYs gained**	**Costs (excluding caregivers) $**	**$**
	
**Slope parameter (τ), which is inversely proportional to the rate of decline in the risk of AMD after quitting relative to current-smokers**			
Upper 95% confidence Limit (slower decline)	1,600	-774,000	391
Lower 95% confidence Limit (faster decline)	1,623	-1,426,000	Dominant
**Higher utilities for reduced visual acuity^¶^**	1,600	-1,082,000	199
**Ranibizumab treatment of neovascular AMD^||^**			
Base-case scenario, as in Table 3, but:			
low ranibizumab cost	1611	-360,000	645
50% of neovascular patients treated	1613	-732,000	414
Sustained-effect scenario, low ranibizumab cost	1610	-282,000	694
Non-sustained effect scenario, high ranibizumab cost	1611	-929,000	292

* Censored at age 85 years

^† ^AMD: Age-related macular degeneration

^‡ ^From the U.S. population of smokers in 2004–2005[10]

^§^Costs are in 2004 U.S. dollars and were rounded. Costs, blind-years and QALYs were discounted at 3% per annum

¶Source: Brown et al., estimated with standard gamble method.[25] For 30 letters read, for example, utility = 0.71, rather than 0.52 in the base case.

|| Base-case, Sustained-effect and Non-sustained effect scenarios as defined in previous paper.[7] Low ranibizumab cost = bevazicumab price ($50 per dose). High ranibizumab price = wholesale price ($1,950 per dose).

## Discussion

The 2004 U.S. Surgeon General's report concluded that the available evidence was suggestive of a causal relationship between smoking and both neovascular and atrophic age-related macular degeneration,[31] and summarized 3 biologic mechanisms whereby smoking might exacerbate or accelerate the degenerative changes that occur in the macula with age. A subsequent review, that included 5 additional studies, confirmed a strong association between current smoking and age-related macular degeneration which fulfilled accepted causality criteria, and concluded that there was evidence of reversibility.[1] We quantified the reduction in risk over time since quitting smoking, using data from 7 studies involving 1,488 people with age-related macular degeneration. [14-20] Although none of the studies monitored the incidence of macular degeneration prospectively in ex-smokers from the time of quitting, all studies found a reduced risk 10 years or more after ex-smokers reported having quit.

We assessed the benefits of smoking cessation in terms of the reduced incidence, morbidity and direct costs of age-related macular degeneration experienced by ex-smokers compared with smokers. In order to concentrate attention on macular degeneration, the numerous other benefits associated with quitting, such as morbidity reductions and health care cost savings associated with lower risks of myocardial infarction, stroke, lung cancer and chronic obstructive pulmonary disease, were not considered.[4] The reduction in all causes mortality risk consequential to quitting was incorporated into the model in order to accurately estimate the macular degeneration-related QALY gain associated with smoking cessation, but gains in life expectancy were not estimated. Our model predicted that smoking cessation was cost-effective even when only its impact on macular degeneration and mortality were considered. This finding was robust to all plausible variations in the model parameter estimates. Even assuming the slowest rate of decline in the risk of age-related macular degeneration after quitting, the incremental cost per QALY gained in 1,000 randomly selected smokers who quit was only $391.

Many effective interventions are available to encourage and assist smokers to quit. These include clinical treatments and services, such as counseling and pharmacotherapies,[32] population-based interventions, such as mass-media anti-smoking advertising and telephone support (quit lines),[33] and policies, such as increasing the price of tobacco products or smoking bans and restrictions.[34] We based our analysis on an estimated cost per quitter for the comprehensive Massachusetts Tobacco Control Program, which comprised treatment services, a mass media campaign, a tobacco surcharge and other local policies.[6] A wide range of costs per quitter have been reported for smoking cessation interventions, reflecting differences in the efficiency of interventions as well as differences in evaluation methodology.[35] A recent review standardized evaluations of clinical interventions, by adjusting cost-effectiveness ratios to reflect a societal perspective and comply with guidelines for economic evaluation.[36] Of the treatments considered, nicotine replacement therapy plus counseling, compared with counseling alone, had the highest incremental cost per quitter. The average adjusted cost per quitter in 4 studies was $9,100 in 2002 U.S. dollars. Much lower cost-effectiveness ratios have been reported for population-based interventions and policies. For example, adjusted costs per quitter of $298 – $1,593 (1997 U.S. dollars) for mass media education campaigns to promote smoking cessation were calculated by the Task Force on Community Preventive Services,[33] smoke-free workplace policies were found to cost $799 per quitter (2002 U.S. dollars),[37] and the American Cancer Society's telephone counseling service cost $1,300 per quitter (2000 U.S. dollars). All these cost-effectiveness ratios are considerably lower than the threshold cost we calculated of $77,000 per quitter, above which the incremental cost per QALY associated with smoking cessation exceeds $50,000. This indicates that our finding that smoking cessation is cost-effective in terms of its impact on macular degeneration alone is also robust to plausible variation in the cost per quitter, and can be generalized to most, if not all, tobacco control strategies.

In a previous paper, we assessed the cost-effectiveness of ranibizumab, a new therapy for the most common type of age-related macular degeneration.[7] Ranibizumab is the first treatment for age-related macular degeneration that improves visual acuity and its efficacy has been described as miraculous.[38] Over time horizons of 2 to 10 years we found that ranibizumab had incremental costs per QALY that would support description of the treatment as "cost-effective".[7] Health care funding bodies have also concluded that ranibizumab's cost-effectiveness is acceptable. It has been recommended by NICE in the United Kingdom,[39] and subsidized by the Australian government under its Pharmaceutical Benefits Scheme.[40] In this paper, we assessed the cost-effectiveness of smoking cessation as a strategy to prevent macular degeneration over a different time period from the ranibizumab analysis – the remaining lifetime of a quitter, censored at age 85 – because the benefits of quitting accrue gradually over a long time period. We found that smoking cessation was unequivocally cost-effective in terms of age-related macular degeneration outcomes. Our model predicted gains in QALYs, and savings in the cost of macular degeneration treatment and the cost of care for people with impaired vision. Our findings have two potential practical applications. First, they will hopefully prompt ophthalmologists, who prescribe ranibizumab, to encourage patients to quit smoking in the interests of their sight. Second, our analysis will provide tobacco control advocates seeking government funding for anti-smoking programs with evidence that such programs are as justifiable on cost-effectiveness grounds as the newly available treatment for macular degeneration.

## Conclusion

This analysis strongly supports the implementation of smoking cessation interventions to prevent age-related macular degeneration, because of their unequivocal cost-effectiveness.

## Competing interests

The authors declare that they have no competing interests.

## Authors' contributions

All authors participated in designing the study. SFH sourced the data. JPM programmed and ran the Markov model. SFH drafted the manuscript, and all authors participated in critically revising the manuscript and approved the final version.

## References

[B1] Thornton J, Edwards R, Mitchell P, Harrison RA, Buchan I, Kelly SP (2005). Smoking and age-related macular degeneration: a review of association. Eye.

[B2] Smith W, Assink J, Klein R, Mitchell P, Klaver CC, Klein BE, Hofman A, Jensen S, Wang JJ, de Jong PT (2001). Risk factors for age-related macular degeneration: Pooled findings from three continents. Ophthalmology.

[B3] Parrott S, Godfrey C (2004). Economics of smoking cessation. BMJ.

[B4] Hurley SF, Matthews JP (2007). The Quit Benefits Model: a Markov model for assessing the health benefits and health care cost savings of quitting smoking. Cost Eff Resourc Alloc.

[B5] Hurley SF, Matthews JP (2008). Cost-effectiveness of the Australian National Tobacco Campaign. Tob Control.

[B6] Biener L, Harris JE, Hamilton W (2000). Impact of the Massachusetts tobacco control programme: population based trend analysis. BMJ.

[B7] Hurley SF, Matthews JP, Guymer RH (2008). Cost-effectiveness of ranibizumab for neovascular age-related macular degeneration. Cost Eff Resour Alloc.

[B8] (2006). TreeAge Pro 2006 User's Manual.

[B9] Hussain B, Saleh GM, Sivaprasad S, Hammond CJ (2006). Changing from Snellen to LogMAR: debate or delay?. Clin Experiment Ophthalmol.

[B10] (2007). Smoking Status by Age, Sex, and Race/Ethnicity: United States, 1997–2005. National Health Interview Survey (NHISS05). National Center for Health Statistics Centers for Disease Control.

[B11] Gold MR, Siegel JE, Russell LB, Weinstein MC, eds (1996). Cost-effectiveness in health and medicine.

[B12] Klein R, Klein BE, Jensen SC, Meuer SM (1997). The five-year incidence and progression of age-related maculopathy: the Beaver Dam Eye Study. Ophthalmology.

[B13] Tomany SC, Wang JJ, van Leeuwen R, Klein R, Mitchell P, Vingerling JR, Klein BE, Smith W, de Jong PT (2004). Risk factors for incident age-related macular degeneration: pooled findings from 3 continents. Ophthalmology.

[B14] Christen WG, Glynn RJ, Manson JE, Ajani UA, Buring JE (1996). A prospective study of cigarette smoking and risk of age-related macular degeneration in men. JAMA.

[B15] Seddon JM, Willett WC, Speizer FE, Hankinson SE (1996). A prospective study of cigarette smoking and age-related macular degeneration in women. JAMA.

[B16] Evans JR, Fletcher AE, Wormald RP (2005). 28,000 Cases of age related macular degeneration causing visual loss in people aged 75 years and above in the United Kingdom may be attributable to smoking. Br J Ophthalmol.

[B17] Khan JC, Thurlby DA, Shahid H, Clayton DG, Yates JR, Bradley M, Moore AT, Bird AC (2006). Smoking and age related macular degeneration: the number of pack years of cigarette smoking is a major determinant of risk for both geographic atrophy and choroidal neovascularisation. Br J Ophthalmol.

[B18] Vingerling JR, Hofman A, Grobbee DE, de Jong PT (1996). Age-related macular degeneration and smoking. The Rotterdam Study. Arch Ophthalmol.

[B19] Delcourt C, Diaz JL, Ponton-Sanchez A, Papoz L (1998). Smoking and age-related macular degeneration. The POLA Study. Arch Ophthalmol.

[B20] McCarty CA, Mukesh BN, Fu CL, Mitchell P, Wang JJ, Taylor HR (2001). Risk factors for age-related maculopathy: the Visual Impairment Project. Arch Ophthalmol.

[B21] Breslow NE, Day NE (1987). IARC Sci Publ.

[B22] Steinbrook R (2006). The price of sight – ranibizumab, bevacizumab, and the treatment of macular degeneration. N Engl J Med.

[B23] Halpern MT, Schmier JK, Covert D, Venkataraman K (2006). Resource utilization and costs of age-related macular degeneration. Health Care Financ Rev.

[B24] (2007). U.S. Medical Care. Consumer Price Index. USDepartment of Labour Bureau of Labour Statistics.

[B25] Brown GC, Sharma S, Brown MM, Kistler J (2000). Utility values and age-related macular degeneration. Arch Ophthalmol.

[B26] Miniño AM, Heron M, Murphy SL, Kochanek KD (2007). Natl Vital Stat Rep.

[B27] Taylor DH, Hasselbad V, Henley J, Thun MJ, Sloan FA (2002). Benefits of Smoking Cessation for Longevity. Am J Public Health.

[B28] Behrendt C (2004). Visual acuity and its decrease in classic neovascular age-related macular degeneration. Ophthalmic Epidemiol.

[B29] (2007). Population Estimates for the U.S., Regions, and States by Selected Age Groups and Sex: Annual Time series, July 1, 1990 to July 1, 1999 (includes revised April 1, 1990 population counts). US Census Bureau.

[B30] (2007). Consumer Price Index – All Urban Consumers. U.S. City Average. All items. USDepartment of Labor Bureau of Labor Statistics.

[B31] (2004). The health consequences of smoking: a report of the Surgeon General.

[B32] (2000). Clinical Practice Guideline Treating Tobacco Use and Dependence.

[B33] Hopkins DP, Briss PA, Ricard CJ, Husten CG, Carande-Kulis VG, Fielding JE, Alao MO, McKenna JW, Sharp DJ, Harris JR (2001). Reviews of evidence regarding interventions to reduce tobacco use and exposure to environmental tobacco smoke. Am J Prev Med.

[B34] Ranson K, Jha P, Chaloupka FJ, Nguyen S, Jha P, Chaloupka F (2002). The effectiveness and cost-effectiveness of price increases and other tobacco-control policies. Nicotine Tob Res.

[B35] Warner KE (1997). Cost effectiveness of smoking-cessation therapies. Interpretation of the evidence and implications for coverage. Pharmacoeconomics.

[B36] Ronckers ET, Groot W, Ament AJ (2005). Systematic review of economic evaluations of smoking cessation: standardizing the cost-effectiveness. Med Decis Making.

[B37] Ong MK, Glantz SA (2005). Free nicotine replacement therapy programs vs implementing smoke-free workplaces: a cost-effectiveness comparison. Am J Public Health.

[B38] Stone EM (2006). A very effective treatment for neovascular macular degeneration. N Engl J Med.

[B39] (2008). Final appraisal determination. Ranibizumab and pegaptanib for age-related macular degeneration. National Institute for Health and Clinical Excellence.

[B40] Australian Government Department of Health and Ageing (2007). New PBS listings for the treatment of Age-related Macular Degeneration. PBS for health professionals.

